# Definition and classification for adverse events following spinal and peripheral joint manipulation and mobilization: A scoping review

**DOI:** 10.1371/journal.pone.0270671

**Published:** 2022-07-15

**Authors:** Martha Funabashi, Lindsay M. Gorrell, Katherine A. Pohlman, Andrea Bergna, Nicola R. Heneghan

**Affiliations:** 1 Division of Research and Innovation, Canadian Memorial Chiropractic College, Toronto, ON, Canada; 2 Department of Chiropractic, Université du Québec à Trois-Rivières, Trois-Rivières, QC, Canada; 3 Department of Chiropractic Medicine, Integrative Spinal Research Group, University of Zürich and University Hospital Balgrist, Zürich, Switzerland; 4 Research Center, Parker University, Dallas, TX, United States of America; 5 Research Department, SOMA Istituto Osteopatia Milano, Milan, Italy; 6 AISO-Associazione Italiana Scuole di Osteopatia, Pescara, Italy; 7 School of Sport, Exercise & Rehabilitation Sciences, University of Birmingham, Birmingham, United Kingdom; Kaohsuing Medical University Hospital, TAIWAN

## Abstract

**Introduction:**

Spinal and peripheral joint manipulation and mobilization are interventions used by many healthcare providers to manage musculoskeletal conditions. Although there are many reports of adverse events (or undesirable outcomes) following such interventions, there is no common definition for an adverse event or clarity on any severity classification. This impedes advances of patient safety initiatives and practice. This scoping review mapped the evidence of adverse event definitions and classification systems following spinal and peripheral joint manipulation and mobilization for musculoskeletal conditions in adults.

**Methods:**

An electronic search of the following databases was performed from inception to February 2021: MEDLINE, EMBASE, CINAHL, Scopus, AMED, ICL, PEDro, Cochrane Library, Open Grey and Open Theses and Dissertations. Studies including adults (18 to 65 years old) with a musculoskeletal condition receiving spinal or peripheral joint manipulation or mobilization and providing an adverse event definition and/or classification were included. All study designs of peer-reviewed publications were considered. Data from included studies were charted using a standardized data extraction form and synthesised using narrative analysis.

**Results:**

From 8248 identified studies, 98 were included in the final synthesis. A direct definition for an adverse event and/or classification system was provided in 69 studies, while 29 provided an indirect definition and/or classification system. The most common descriptors to define an adverse event were causality, symptom severity, onset and duration. Twenty-three studies that provided a classification system described only the end anchors (e.g., mild/minor and/or serious) of the classification while 26 described multiple categories (e.g., moderate, severe).

**Conclusion:**

A vast array of terms, definition and classification systems were identified. There is no one common definition or classification for adverse events following spinal and peripheral joint manipulation and mobilization. Findings support the urgent need for consensus on the terms, definition and classification system for adverse events related to these interventions.

## Introduction

Spinal pain, including low back and neck pain, is the most common musculoskeletal problem globally, a leading cause of disability and absenteeism from work and is ever increasing [1]. These factors contribute to increased socioeconomic burdens and costs [2]. Clinical guidelines and best practice recommendations (e.g., NICE Guidelines) advocate the use of conservative interventions, including spinal and peripheral joint manipulation and mobilization, provided by a variety of healthcare professionals (e.g., chiropractors, naprapaths, osteopaths, physiotherapists, physicians, *etc*.) as a management option for uncomplicated presentations of spinal pain [3–5]. Used globally by manual therapists as conservative interventions, spinal and peripheral joint manipulation involves the application of a high-velocity, low-amplitude force to a specific joint, whilst spinal and peripheral joint mobilization involves the application of a cyclic low-velocity force [6].

Similar to any medical intervention, joint manipulations and mobilizations are not without risk of harms or complications [7]. Whilst serious harms have been reported to be rare [8–11], the consequences of such can be devastating, with considerable impact on those involved. Patient safety remains a top priority within healthcare, with a continued focus on preventing and minimising adverse events following any type of intervention [12, 13]. A 2015 North American Patient Safety Foundation expert panel emphasised the importance of patient safety as a public health issue with a main recommendation being the need for a common set of safety metrics for use across all practice settings, including primary or ambulatory care settings, which is where the majority of care is provided [14, 15].

“Harms”, “complications”, “side-effects” and “adverse events” are among several commonly used terms in the literature describing undesirable outcomes of manual interventions (e.g., spinal and peripheral joint manipulation and mobilization), which are most commonly used to reduce pain and disability in patients with musculoskeletal complaints [16–18]. Additionally, how these outcomes are defined and what constitutes an adverse event (or undesirable outcome) following spinal and peripheral joint manipulation and mobilization remains disparate [19–21]. These outcomes may be further classified according to their severity (e.g., mild, moderate, severe), onset (e.g., during treatment, within 24–48 hours after treatment), duration (e.g., transient, short-lasting, permanent) or need for unplanned additional remedial or medical care (e.g., investigations, specialist referral, hospitalisation) [22, 23]. The kaleidoscope of domains and descriptors used in the literature to report and characterize these outcomes impedes attempts to advance patient safety initiatives and practices through a common and universal understanding of observed safety incidents. Although previous studies have highlighted this issue [16] and proposed frameworks for categorizing adverse events following manual therapy [23–25], there is still no standardization as to what constitutes an adverse event following such manual therapy interventions. A standardized and accepted adverse event typology would not only facilitate the development of strategies to minimise or prevent such events across all manual therapy professions that use these interventions, but more importantly, achieve consistency and precision in documenting and reporting such events. Specifically, an adverse events typology should include an operational definition of an adverse event so that identification, reporting and learning opportunities can be standardized across professions using spinal and peripheral joint manipulation and mobilization.

For these reasons, a scoping review of the literature is required. Combining the published knowledge from different professions, healthcare settings and musculoskeletal conditions will elucidate the current landscape and true extent of the problem. Findings from this scoping review will provide the evidence needed to conduct further research and move towards a consensus on the topic of adverse events. Ultimately, enhancing patient safety practices for spinal and peripheral joint manipulation and mobilization.

### Aim and objectives

This scoping review aimed to map the scientific literature defining adverse events and their respective classification systems following spinal or peripheral joint manipulation and mobilization for musculoskeletal conditions in an adult population. Specific objectives included:

1. To describe how adverse events following spinal and peripheral joint manipulation and mobilization have been defined in the literature;
2. To describe how adverse events following spinal and peripheral joint manipulation and mobilization have been classified in the literature.

## Materials and methods

### Design

This scoping review followed the Preferred Reporting Items for Systematic reviews and Meta-Analyses extension for Scoping Reviews (PRISMA-ScR) for transparency in reporting [26]. The protocol was registered at the Open Science Framework Registry (10.17605/OSF.IO/UBX2D) and designed by an international, interprofessional team of manual therapists (chiropractors, osteopaths and physiotherapists) with relevant clinical and methodological expertise. A scoping review was chosen as this study focuses on examining and clarifying definitions and classification systems for adverse events following spinal and peripheral joint manipulation and mobilization that are used in the literature [27].

### Stages

This review was conducted in 5 stages: (1) identifying the research question; (2) identifying relevant studies; (3) study selection; (4) charting the data; and (5) collating, summarizing and reporting the results [28, 29]. The optional consultation exercise (step 6) was not included within the scope of this specific manuscript as the results will be used to inform an e-Delphi study [30].

#### Stage 1: Identifying the research question

How does the scientific literature define adverse events and their respective classification systems for events that occur following spinal or peripheral joint manipulation and mobilization for musculoskeletal conditions in an adult population?

#### Stage 2: Identifying relevant studies

*Information sources*. The following databases were searched from inception to 12^th^ February 2021: MEDLINE, EMBASE, CINAHL, Scopus, AMED, ICL, PEDro and Cochrane Library. Grey literature using Open Grey and Open Access Theses and Dissertations (OATD) were also searched.

*Search strategy*. The search strategy was designed by the authors with the assistance of an experienced health sciences librarian. The initial search strategy (S1 Table) was developed for Ovid MEDLINE using medical subject headings (MeSH) and text words. This was subsequently adapted to the syntax and subject headings of the other databases that were searched.

*Eligibility criteria*. Studies were identified by using the eligibility criteria outlined in Table 1.

**Table 1:** Inclusion and exclusion criteria.

Category	Inclusion Criteria	Exclusion Criteria
Language	▪ English, Portuguese, Italian	
Participants	▪ Adults (average age 18 to 65 years old)	▪ Pediatric population (average age < 18 years old)
	▪ Primary musculoskeletal condition (e.g., back pain, neck pain, cervicogenic / tension-type headache, temporomandibular joint pain, *etc*.)	▪ Older adults (average age > 65 years old)
	▪ Secondary musculoskeletal condition (e.g., shoulder pain due to surgery/cancer, *etc*.)	▪ Non-musculoskeletal conditions (e.g., neurological conditions [e.g., migraine, stroke, *etc*.], pulmonary, cardiac, rheumatological conditions, *etc*.)
		▪ Participants seeking medical management for a musculoskeletal condition (e.g., fracture, arthroplasty, *etc*.)
Intervention	▪ Spinal or peripheral joint manipulation or mobilization used as the primary intervention (i.e., forces applied directly to skin overlying a joint)	▪ Pharmacological or surgical interventions
		▪ Other interventions, such as active interventions (e.g., exercise, active stretching, *etc*.), ancillary procedures (e.g., taping, heat, ice, ultrasound, laser, *etc*.), other manual therapy modalities (e.g., massage therapy, acupressure, soft tissue therapy, *etc*.), as primary interventions, with manipulation or mobilization being secondary
	▪ Multimodal intervention including manipulation or mobilization (e.g., exercise + manipulation or mobilization, *etc*.)	
		▪ Indirect joint manipulation or mobilization (e.g., fascial mobilization, muscle energy technique, *etc*.)
		▪ Manipulation under anesthesia
		▪ Early mobilization (e.g., Continuous Passive Movement, early mobilization after surgery, mobilization of Intensive Care Unit patients, *etc*.)
		▪ Manipulation or mobilization with a mechanical device (e.g., Activator™, Impulse™, robotic manipulation or mobilization, Cox tables, *etc*.)
Outcomes	▪ Adverse events definition	▪ Adverse event report, but without an adverse event definition
	▪ Classification (i.e., symptom severity, onset, duration, need for unplanned additional remedial or medical care, *etc*.)	▪ Adverse event reports, but with no mention of manipulation or mobilization
Study design	▪ Peer-reviewed publication (e.g., literature reviews, meta-analyses, clinical practice guidelines, experimental studies, clinical studies, qualitative studies, observational studies, surveys, case series and reports, study protocols, *etc*.)	▪ Editorials, conference proceedings, commentaries, letter to the editor, expert opinion, secondary sources (e.g., textbooks, *etc*.)
		▪ Case reports that do not mention the terms “adverse event”, “complication”, “side effect”, “adverse reaction”, *etc*. or do not imply the condition being an adverse event to manipulation or mobilization

For the purpose of this study, mobilization was defined as a manual therapy technique comprising a continuum of skilled passive movements that were applied at varying speeds and amplitudes to joints [6]. Manipulation was defined as a passive, high velocity, low amplitude thrust applied to a joint complex within its anatomical limit (the range of motion of the joint complex in which active and passive motion occurs and not beyond the joint’s anatomic limit) [6]. The term “adverse event” was adopted as an umbrella term to reflect any undesirable effect of spinal or peripheral joint manipulation and mobilization where terms such as “harms”, “complications”, “side-effects”, *etc*. have also been used in the literature [16–18].

#### Stage 3: Study selection

This stage was conducted in 2 phases with each phase starting with a pre-screening team meeting to discuss inclusion and exclusion criteria. Both phases were performed using Covidence systematic review software (Veritas Health Innovation, Melbourne, Australia), an online tool developed for systematic reviews by the Cochrane Collaboration that follows the Preferred Reporting Items for Systematic reviews and Meta-Analyses (PRISMA) guidelines. Five reviewers screened the same 20 publications to ensure inter-screener calibration with weekly meetings to resolve any conflicts. Specifically, in phase 1, titles and abstracts were independently screened by two of the five reviewers to identify potentially relevant studies. Any disagreements were resolved through discussion. Phase 2 was based on the full texts of all studies identified as potentially relevant during phase 1. Similar to phase 1, two of the five reviewers independently screened full texts and any disagreements were resolved by discussion and consensus with all five reviewers.

#### Stage 4: Charting the data

Data were extracted by all five reviewers working as a group using a standardized data extraction form that was first piloted with 20 included studies. All data extracted were checked for accuracy by two reviewers (MF and LG). Disagreements were resolved through discussion and consensus.

Extracted data included study characteristics (first author, year of publication, title, country, study design [original studies: case report/series, observational studies, consensus, survey, experimental trials, other designs; and clinical practice guidelines, review or study protocols]), participant population for original studies or study protocol (sample size, condition being treated [spinal, peripheral, mixed, unknown, not applicable], condition severity, condition chronicity [acute (<3 weeks), subacute (>3 weeks, <3 months), chronic (>3 months), mixed, unknown]), studies description for clinical practice guidelines and review (number of included studies, design of included studies), intervention characteristics (setting [community-based clinic/office, hospital, research clinic, academic institute, mixed], profession [chiropractic, naprapathy, osteopathy, physiotherapy, mixed, other, unknown], intervention [manipulation, mobilization, mixed]), and adverse event characteristics (definition, classification system [e.g., minor-moderate-major; mild-moderate-severe-serious], citations for adverse event definitions or classification systems, and whether the provided adverse event definition was direct (a clear statement of what was considered an adverse event) or indirect (indicated what was considered an adverse event without a clear statement [e.g., provided the question asked to participants during the study])).

#### Stage 5: Collating, summarizing and reporting the results

Extracted data were categorized into two groups: i) studies providing a direct definition and/or classification system for adverse events following spinal and peripheral joint manipulation or mobilization; and ii) studies providing an indirect definition and/or classification system for adverse events following these interventions. Specifically, studies providing a direct definition and/or classification were those that provided a clear statement of the study’s operational definition and/or classification system for adverse events. For example: “For the purposes of this study, we adopted the following definition (derived from the ICH Guidelines for Good Clinical Practice): *An adverse event (AE) can be any unfavourable and unintended sign*, *symptom*, *or disease temporally associated with the use of an intervention (treatment)*, *which does not necessarily have a causal relationship with such treatment*” [31]. Studies providing an indirect definition and/or classification were those that did not provide a clear statement of their operational definition and/or classification system, but indicated what was considered an adverse event, for example, by providing the question used to collect adverse events in a survey. For example: “[…] possible adverse effects were assessed by 2 open-ended questions: (1) “Did your symptoms get worse after this treatment?” and (2) “Are you feeling any different symptoms after this treatment?” [32]. A descriptive summary detailing the overall number of studies included in the review, their study characteristics as well as the data regarding adverse event definition and classification system extracted from included studies are provided.

## Results

### Study selection

**Fig 1:**
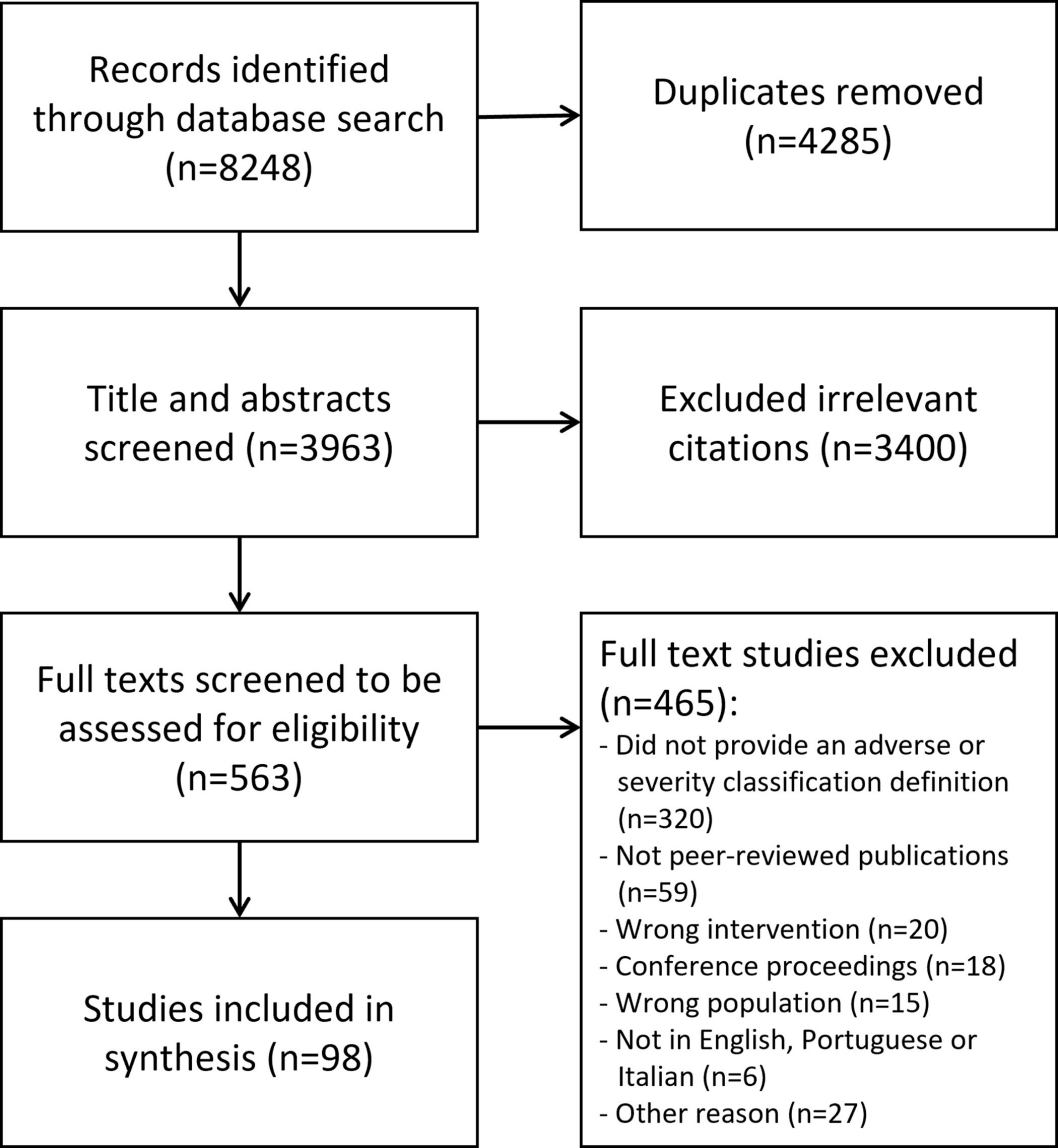
PRISMA Sc-R flowchart.

Electronic searches identified 8248 citations that resulted in 3963 unique citations to be screened for inclusion following the removal of duplicates. The titles and abstracts were assessed for their relevance to the review based on the eligibility criteria (phase 1 screening), where 3400 citations were excluded, resulting in 563 citations for full text review. The phase 2 screening excluded 465 full texts: 320 did not provide an adverse event definition or classification, 59 were not peer-reviewed publications, 20 included the wrong intervention (e.g., did not use joint manipulation or mobilization), 18 were conference proceedings, 15 included the wrong population (e.g., participants younger than 18 or older than 65 years old), 6 were not written in English, Portuguese or Italian and 27 were excluded for other reasons (e.g., full text not available, professional issue papers, *etc*.). As such, 98 studies were included in this scoping review (Fig 1).

### Characteristics of included studies

The 98 included studies were published between 1993 and 2021, mostly from North America (n = 42) and Europe (n = 36). Thirty-three studies focused on joint manipulation, 5 focused on mobilization and 60 included both techniques. Study settings mainly comprised academic institutes (n = 40) and provided manipulation or mobilization to the spine (n = 67). Study designs were: literature reviews (n = 21), surveys (n = 20), clinical trials (n = 18), observational studies (n = 10), protocols (n = 10), case report/series (n = 6), consensus studies (n = 5), clinical practice guidelines (n = 4), qualitative studies (n = 2), and “other” (n = 2) (e.g., retrospective analysis). A direct definition for an adverse event and/or classification system was provided in 69 studies, while 29 provided an indirect definition and/or classification system.

### Data synthesis

#### Studies with a direct adverse event definition and/or classification system

The studies that provided a direct definition of an adverse event and/or classification system (n = 69) were published between 1993 and 2020 and were mostly from North America (n = 28) and Europe (n = 27). Twenty-two studies focused on joint manipulation, 4 focused on mobilization and 43 included both techniques. Most were conducted in an academic institute (n = 26) and provided the intervention to the spine (n = 48).

Study designs included: surveys (n = 15), reviews (n = 13), trials (n = 12), protocols (n = 9), observational studies (n = 6), consensus studies (n = 5), case reports/series (n = 4), clinical practice guidelines (n = 2) and other designs (e.g., qualitative studies) (n = 3). Of these, 19 studies provided a direct definition of an adverse event only, 20 provided a classification system only, and 30 provided both a direct definition and classification system (Tables 2–4).

**Table 2:** Studies providing direct adverse event definition and classification system (n = 30).

Author, year, citation	Study Design	Definition	Classification system
**Powell et al. 1993** [8]	Case Report / series	The definition of "complication" is broadened to include indirect injury in which treatment postpones the appropriate diagnosis or continues in spite of progressive symptoms or signs, then the incidence may be higher.	**Severe**: if the treatments were unsuccessful, resulted in major disability or death.
**Senstad et al. 1996a** [36]	Survey	Some type of unpleasant side effect after the previous treatment	**Adequate reactions**: does not influence the working ability with spontaneous remission completed, at the latest, 2 days after the manipulation
			**Exceeding reactions**: objective worsening of the pre-existing state, with decreased work capacity, and spontaneous remission exceeding 2 days
			*Authors used*:
			On a scale from 1 to 4:
			**Minor** = 1
			**Unbearable** = 4
**Leboeuf-Yde et al. 1997** [37]	Survey	Any additional discomfort after the previous treatment	**Common/Normal reactions**: early onset, mild-to-moderate severity and gone within 48 hours
		Self-reported unpleasant reactions	**Light or moderate reactions**: little or no effect on normal daily activities
**Thiel et al. 2006** [38]	Survey	A patient safety incident can be defined by:	**Low Harm**: Incident required extra observation or minor treatment (additional therapy or medication over short period of time, does not include admission to hospital or attending as an outpatient on repeated occasions)
			**Moderate Harm**: Incident resulted in a moderate increase in additional treatment (admission to hospital or attending as an outpatient on repeated occasions, or requiring surgery or prolonged episodes of care) and caused significant but not permanent harm
		‘‘That was a threat to my patient’s well-being, and I don’t want it to happen again”	**Severe Harm**: Incident resulted in permanent harm (harm or disability directly related to incident and leading to permanent lessening of bodily functions, or sensory, motor, physiologic or intellectual deficit)
			**Death**: Incident directly resulted in death
			**Near Miss**: No harm occurred—Incident had potential to cause harm but was prevented either by chance or deliberate action
**Rubinstein et al. 2007** [9]	Observational Study	A new related complaint that was not present at baseline or a worsening of the presenting complaint or an existing complaint by >30% compared with baseline	**Intense AE**: any AE fulfilling our definition of an adverse event and that also scored ≥8 in intensity on the 11-point NRS.
			**Serious AE**: events resulting in death, life-threatening situations, the
			need for admittance to a hospital, or temporary or permanent disability.
**Rubinstein et al. 2008** [39]	Observational Study	A new related complaint that was not present at baseline, or a worsening of the neck pain or any other existing complaint by >30% compared to baseline.	**Intense AE**: any AE fulfilling our definition of an adverse event, and which also scored ≥8 in intensity on the 11-point NRS.
			**Serious AE**: events resulting in death, life-threatening situations, the need for admittance to a hospital, or temporary or permanent disability.
**Rubinstein et al. 2008** [40]	Observational Study	Either a new complaint or the worsening of an existing complaint by more than 30% compared to baseline (more than MCID on 11-point NRS)	**Intense AE**: any AE fulfilling the definition, and which also scored 8 or higher in intensity on the 11-point NRS.
			**Serious AE**: events resulting in death, life-threatening situations, the need for admittance to a hospital, or temporary or permanent disability.
**O’Shaughnessy et al. 2010** [35]	Case Report / series	Side-effects of a benign nature included increased pain and/or stiffness of short duration	**Mild to moderate**: little or no influence on daily activities, brief, with spontaneous recovery and lasting no more than a few days.
			**Major complications**: irreversible
**Wangler et al. 2011** [41]	Consensus	An adverse event is an injury related to medical management, in contrast to a complication of disease.	Preventable or Non-preventable.
		Adverse drug reaction is a complication that occurs when the medication is used as directed and in the usual dosage.	
			**Preventable AE**: An adverse event caused by an error or other type of systems or equipment failure.
		An adverse event is the result of a care delivery problem related to chiropractic management, in contrast to complications of disease.	
**Eriksen et al. 2011** [42]	Trial	Symptomatic Reaction: a new related complaint that was not present at baseline or a worsening of the presenting complaint by >30% compared with baseline occurring <24 hours following care	**Intense symptomatic reaction**: any complaint fulfilling our definition of a SR that also scored ≥8 in intensity on the 11-point NRS
		Any discomfort or unpleasant reaction that they felt was related to their chiropractic care	**Serious AE**: events resulting in death, life-threatening situations, need for admittance to a hospital, or temporary or permanent disability
**Puentedura et al. 2011** [43]	Trial	Sequelae medium- to long-term in duration, with moderate to severe symptoms that were serious, distressing, and unacceptable to the patient and required further treatment	**Minor side effects**: short term, mild in nature, nonserious, transient, were reversible consequences of the treatment and disappear within 24 to 48 hours
**Rubinstein et al. 2011** [44]	Review	Transient increase in pain; exacerbations of symptoms; aggravation of condition, worsening of pain	**Serious AE**: defined as an event leading to hospitalisation or death within one week of treatment
**Dagenais et al. 2012** [45]	Survey	Harm is any negative health outcome observed in a clinical trial, regardless of whether it was definitively related to the intervention. This is true even when the harm does not become apparent until the treatment has been used by many patients for extended periods. This term, therefore, incorporates concepts related to adverse events, adverse reactions, side effects, and safety, and favors disclosure regardless of causation.	**Benign**: mild to moderate intensity, and resolve spontaneously
			**Minor**: short term, mild intensity and do not require treatment; gets better on their own in a few days
			**Moderate**: medium to long term and of moderate intensity
			**Major**: medium to long term, require treatment and are of moderate to severe intensity
			**Serious**: result in death, hospitalization, or permanent injury
**Nee et al. 2012** [46]	Trial	Aggravation of existing symptoms or provocation of other unpleasant sensations after each neural tissue management treatment session	**Mild**: do not require additional treatment, usually last <24 hours, have minimal impact on daily activities, and do not reduce a participant’s chance of improving with neural tissue management
**Walker et al. 2013** [47]	Trial	From protocol:	On 0–10 NRS score:
			**Mild** = 1 to 3
		Adverse events are undesirable reactions to treatment.	
			**Moderate** = 4 to 6
		Any new unwelcome symptoms OR an increase of your presenting symptoms during the first 48 hours (two days) after treatment	**Severe** = 7 to 10
**Pohlman et al. 2014** [22]	Consensus	Any unfavorable sign, symptom, or disease temporally associated with the treatment, whether or not caused by the treatment.	**Mild**: Asymptomatic or mild symptoms, self-care only (e.g., ice/heat, over-the-counter analgesic);
			**Moderate**: Limiting age-appropriate activities of daily living (e.g., work, school) OR sought care from a medical doctor;
			**Severe**: Medically significant but not immediately life-threatening; temporarily limits self-care (e.g., bathing, dressing, eating); OR urgent or emergency room assessment sought;
			**Serious**: Results in death OR a life-threatening adverse event OR an AE resulting in inpatient hospitalization or prolongation of existing hospitalization for more than 24 hours; a persistent or significant incapacity or substantial disruption of the ability to conduct normal life functions; a congenital anomaly/birth defect
**Paanalahti et al. 2014** [19]	Trial	Events that had occurred within 24 hours following the treatment.	Definitions based on duration and/or severity of the reaction:
			**Short minor**: NRS ≤3 and <24 hours of duration
			**Long minor**: NRS ≤3 and ≥24 hours of duration
			**Short moderate**: NRS >3 and <24 hours of duration
			**Long moderate**: NRS >3 and ≥24 hours of duration
			**Serious adverse event**: the patient had a loss of bowel/bladder function, stroke, fracture or where hospitalized
**Dougherty et al. 2014** [20]	Trial	Any undesirable medical event with new onset or significant exacerbation during the course of the study, regardless of whether or not it was considered to be related to study treatment	**Serious**: any AE occurring during the study or within 30 days of conclusion of study participation resulting in any one of the following outcomes: death, life threatening persistent or significant disability/incapacity, hospitalization (when the result of an AE occurring during the study; note, hospitalization for an elective procedure or for treatment of a pre-existing condition not worsened during the study was not considered an serious AE; admission to the emergency room for 23 hours or less was not considered a hospitalization), congenital anomaly, important medical event (i.e., an event that in the opinion of the investigator may jeopardize the participant and may require medical or surgical intervention to prevent one of the outcomes listed above)
**Han et al. 2015** [48]	Protocol	Any new unwelcome symptoms or an increase of presenting symptoms during the first 48 hours (two days) after treatment;	**Mild AE**: 1-3/10 on an 11-point NRS
			**Moderate AE**: Original pain increased regionally or/and radically after the intervention, with its intensity rated below 8/10 on an 11-point NRS (From questionnaire [additional file 1] = 4-6/10 on 11-point NRS)
		Any additional problems or increasing difficulties with the activities of daily living after spinal manipulation	**Severe AE**: the radicular pain intensity is rated above 8/10 on an 11-point NRS, ankle-foot sensorimotor function is suddenly absent, or defecation dysfunction and saddle anesthesia occur (From questionnaire [additional file 1] = 7-10/10 on 11-point NRS)
**Woodfield et al. 2015** [49]	Review	An increase in baseline pain or a new complaint within 24 hours	**Mild** symptomatic reactions of short-duration (<24 hours) and only “little” effect on daily activities, similar to the short-term effect of exercise.
**Kim et al. 2016** [50]	Protocol	Any unexpected signs or symptoms during the treatment	Spilker classification:
			**Mild:** not needing additional intervention, nor significantly inhibiting to the normal lifestyle (function) of the participant
			**Moderate:** significantly inhibiting to the normal lifestyle (function) of the participant, and may need additional intervention, recovering afterwards
			**Severe:** severe AE requiring intensive intervention, and leaving sequelae
**Thoomes-de Graaf et al. 2017** [51]	Survey	Reference to any untoward medical occurrence and the lack of a causal relationship	**Minor adverse events**: short term and mild, nonserious, transient and easily reversible, requiring no further treatment or alteration of management strategy as the consequences are short term and contained.
			**Moderate adverse effects** were defined identically, but being only moderate in severity.
			**Major adverse effects** were defined as lasting medium to long term, being moderate to severe, unacceptable, serious and distressing and normally requiring further treatment.
**Nielsen et al. 2017** [21]	Review	Any untoward occurrence that may present during treatment	**Serious AEs** are conditions requiring hospital admission (or mortality)
**Swait et al. 2017** [11]	Review	Untoward, undesirable or detrimental, have an impact on the patient and are caused by a healthcare process rather than the natural process of disease	**Mild or benign events**: short-term, non-serious, the patient’s function remains intact, and they are transient/reversible; no treatment alterations are required because the consequences are short-term and contained.
			**Moderate AEs**: as major adverse events but moderate in severity.
			**Major AEs**: medium to long-term, moderate to severe and unacceptable; they normally require further treatment and are serious and distressing.
			*Authors used*:
			**Benign**: mild to moderate, transient adverse events
			**Serious**: moderate to major, long-term adverse events
**Shin et al. 2017** [52]	Protocol	Any unexpected or unintended patient reaction	Spilker classification:
			**Mild**: not needing additional intervention, nor significantly inhibiting to the normal lifestyle (function) of the participant
			**Moderate**: significantly inhibiting to the normal lifestyle (function) of the participant, and may need additional intervention, recovering afterwards
			**Severe**: severe AE requiring intensive intervention, and leaving sequelae
**Degenhardt et al. 2018** [10]	Observational Study	Any unfavorable and unintended sign, symptom, or disease temporally associated with the use of a medical treatment or procedure that may or may not be considered related to the medical treatment or procedure.	**Serious AE**: result in death or hospitalization, or caused permanent disability.
		*Authors used*:	
		Responses of worse or much worse were considered to indicate that an adverse event had occurred	
		Exacerbation of the patients’ chief complaints or a new symptom.	
**Do et al. 2018** [53]	Protocol	Undesirable and unintentional signs (i.e., abnormal laboratory test results), symptoms, or disease occurring after treatment, which does not have to have a causal relationship with the treatment	**Mild**: no need for additional procedures and no great interference with the subject’s everyday life (function)
			**Moderate**: significant interference of the subject’s everyday life (function), probable need for additional procedures but followed by resolution after the procedure
			**Severe**: calling for advanced procedure, and leaving sequelae will be applied for evaluation.
**Tabell et al. 2019** [54]	Observational Study	Pain and loss of function with impact on daily living or work	On a 0–10 NRS scale:
			**No AE** = <1
			**Mild AE** = 1–3
			**Moderate/Major AE** = ≥4
**Lim et al. 2019** [55]	Trial	From protocol:	From protocol:
			Spilker classification:
			**Mild**: not needing additional intervention, nor significantly inhibiting to the normal lifestyle (function) of the participant
			**Moderate**: significantly inhibiting to the normal lifestyle (function) of the participant, and may need additional intervention, recovering afterwards
		Any unexpected or unintended patient reaction	
			**Severe**: severe AE requiring intensive intervention, and leaving sequelae
**Pohlman et al. 2020** [56]	Survey	Any unfavorable sign, symptom, or disease temporarily associated with the treatment, whether or not caused by the treatment, specifically any worsened or new symptom.	**Mild**: Mild symptoms, self-care only (e.g., ice/heat, over-the-counter analgesic).
			**Moderate**: Limiting age-appropriate activities of daily living (e.g., work, school) OR sought care from a medical doctor.
		Worsened and new symptoms	
			**Severe**: Medically significant but not immediately life-threatening; temporarily limits self-care (e.g., bathing, dressing, eating) (for 5 years of age and older); OR urgent or emergency room assessment sought.
		A new AE: a symptom that was not noted pre-treatment but was reported post-treatment.	
		A worsening AE: a symptom noted pre-treatment with increased severity post-treatment	**Serious**: Results in death OR a life-threatening AE OR an adverse event resulting in inpatient hospitalization or prolongation of existing hospitalization for more than 24 hour; a persistent or significant incapacity or substantial disruption of the ability.

NRS: numeric rating scale; MCID: minimal clinically important difference; AE: adverse event.

**Table 3:** Studies providing direct adverse event definition only (n = 19).

Author, year, citation	Study Design	Definition
**Senstad et al. 1997** [57]	Survey	Any unpleasant reaction after the previous treatment
**Malone et al. 2002** [58]	Case Report / series	A detrimental result of the treatment:
		Reaction is a slight or clinically insignificant short-lived symptom
		Incident (or irreversible complication) is an unexpected event resulting in serious impairment, injury, or fatality
**Hurwitz et al. 2005** [59]	Trial	Unpleasant reactions they may have had as a result of chiropractic treatment
**Rajendran et al. 2009** [31]	Survey	Any unfavourable and unintended sign, symptom, or disease temporally associated with the use of an intervention (treatment), which does not necessarily have a causal relationship with such treatment
**Kuczynski et al. 2012** [60]	Review	Unintended consequences of treatment
**Puentedura et al. 2012** [61]	Review	The sequelae following a cervical spine manipulation that are medium to long term in duration, with moderate to severe symptoms, and of a nature that was serious, distressing, and unacceptable to the patient and required further treatment
**Bjorklund et al. 2012** [62]	Protocol	“Much worse” or “Very much worse” on the Patient Global Impression of Change Scale
**Reid et al. 2014** [63]	Trial	Any new symptoms after the interventions and if the symptoms persisted for more than 24 hours
**MacPherson et al. 2015** [64]	Survey	Reactions to treatment that they found unexpected or unpleasant
**Rajendran et al. 2015** [65]	Survey	Any unfavourable and unintended sign, symptom, or disease temporally associated with the use of an intervention (treatment), which does not necessarily have a causal relationship with such treatment.
**Puentedura et al. 2015** [66]	Review	The sequelae following thrust joint manipulation to the spine that are medium to long term in duration, with moderate to severe symptoms, and of a nature that is serious, distressing and unacceptable to the patient and requires further treatment
		Unwanted side effect: short term, mild in nature, non-serious, transient and reversible consequences of the treatment
**Petrozzi et al. 2015** [67]	Protocol	A new related complaint which was not present at baseline or previous visit, or a worsening of the presenting complaint
**Bussieres et al. 2016** [68]	Clinical Practice guideline	Medical occurrence temporally associated with the use of a treatment or a medicinal product, but not necessarily causally related.
		Undesirable outcomes
**Kranenburg et al. 2017** [33]	Consensus	Adverse events are unexpected events that occur following an intervention without evidence of causality
**Kranenburg et al. 2017** [69]	Review	The sequelae following a cervical spinal manipulation that are medium to long term in duration, with moderate to severe symptoms, and of a nature that was serious, distressing, and unacceptable to the patient and required further treatment.
		Side effects are defined as short term, mild in nature, nonserious, transient and reversible consequences of the treatment.
**Heneghan et al. 2018** [70]	Survey	Side-effects are reversible, often transient in nature and are a recognized sequelae of thoracic joint manipulation
		Concerning adverse events: there is the potential for life changing consequences
**Coulter et al. 2018** [71]	Review	Any adverse experience during treatment resulting in death, life-threatening adverse experience, hospitalization or prolongation of existing hospitalization, or persistent or significant disability or incapacity’
**Funabashi et al. 2020** [72]	Survey	An unintended response to treatment that may or may not be caused by the treatment
**Heneghan et al. 2020** [34]	Review	An ‘untoward medical occurrence’ in a patient subjected to an intervention
		Side effects are minor, reversible and short lived
		Adverse events are moderate to severe, last longer and importantly may require medical management

**Table 4:** Studies providing direct adverse event classification system only (n = 20).

Author, year, citation	Study Design	Classification system
**Stern et al. 1995** [73]	Case Report / series	A **minor** complication was defined as increasing symptoms as reported by the patient.
		A **major** complication was defined as emergency surgery after the treatment.
**Hendry et al. 2002** [74]	Survey	**Minor**: benign and transient
		**Moderate**: reversible and serious
		**Major / serious**: irreversible
**UK BEAM Trial Team 2004** [75]	Trial	**Serious adverse events**: treatment related events leading to hospital admission or death within one week
**Gibbons et al. 2006** [76]	Other design	**Transient**: begin within 4 hours of receiving treatment and typically resolve within the next 24 hours.
		**Substantive reversible impairment**
		**Serious non-reversible impairment**
**Thiel et al. 2007** [77]	Survey	**Minor adverse events**: worsening of presenting symptoms or onset of new symptoms, immediately, and up to 7 days, after treatment
		**Significant (serious) event**: referred to hospital and/or severe onset/worsening of symptoms immediately after treatment and/or resulted in persistent or significant disability/incapacity.
**Haneline et al. 2009** [78]	Observational Study	**Serious adverse events**: events resulting in death, life-threatening situations, the need for admittance to a hospital, or temporary or permanent disability.
**Carnes et al. 2010a** [23]	Consensus	**‘Mild’ and ‘not adverse’ adverse events**: short term and mild, they are non-serious, the patient’s function remains intact, they are transient/reversible and no treatment alterations are required because the consequences are short term and contained.
		**‘Moderate’ adverse events**: the same as ‘major’ adverse events but only moderate in severity.
		**‘Major’ adverse events**: medium to long term, moderate to severe and unacceptable; they normally require further treatment and are serious and distressing.
**Carlesso et al. 2011** [79]	Other design	**Not Adverse**: short term duration, acceptable severity, intact function and no other explanation possible
		**Mild**: short term (hours to 2 days) duration, 0.5–2 on NRS severity, intact function and no other explanation possible
		**Moderate**: medium term (1–5 days) duration, 1–2 on NRS severity, modified function, no other explanation possible
		**Major**: longer term (>2 days/next visit) duration, >3 on NRS severity, unacceptable symptoms, impaired function, no other explanation possible
**Carlesso et al. 2013** [25]	Survey	**Mild**: acceptable and short-term, no functional impact, lasting up to 2 days
		**Major events**: impacting on function
**Carlesso et al. 2013** [24]	Trial	**Mild**: No impact on function; lasts less than 24 hours
		**Moderate**: Function modified but intact, may require alteration in treatment, lasts between 24 hours to 1 week
		**Major**: Function absent, requires medical intervention, lasts over 1 week
**Hebert et al. 2015** [80]	Review	**Serious adverse event** was defined as an untoward occurrence that results in death or is life threatening, requires hospital admission, or results in significant or permanent disability
**Keating et al. 2015** [81]	Protocol	**Significant adverse event** presents progressive neurological signs; or emerging medical red flags or cervical spondylotic myelopathy
**Kressig et al. 2016** [82]	Other design	**Serious adverse events**: symptoms with immediate onset after treatment and with persistent or significant disability.
**Lisi et al. 2018** [83]	Consensus	**Serious adverse event**: resulting in death, life-threatening symptoms, hospitalization, or disability or requiring intervention to prevent permanent impairment or damage.
**Frydman et al. 2018** [84]	Protocol	**Serious adverse events** are defined as events that are fatal, life-threatening or lead to hospitalisation.
**Smith et al. 2019** [85]	Review	**Serious adverse events** associated with manipulative therapies are defined as conditions that lead to hospital admission, permanent damage or death.
**Yao et al. 2020** [86]	Trial	**Nonserious adverse events**: self-limited, and no permanent injuries occurred
		**Serious adverse events**: caused death, were life threatening, or necessitated admission to the hospital
**Gross et al. 2002** [87]	Clinical Practice guideline	**Minor**: relatively common benign transient side effects, lasting less than 24 hours
		**Moderate**: reversible serious complications
		**Major complications**: irreversible serious complications
**Skelly et al. 2020** [88]	Review	**Minor adverse events**: mild symptoms and time-limited
		**Nonserious treatment-related adverse events**: worsening of symptoms, mild, self-limiting back or joint pain
		**Serious adverse events**: involving death, hospitalization, persistent disability, requiring intensive medical attention or a life-threatening risk
**Funabashi et al. 2020** [89]	Protocol	**Serious adverse events:** any unfavorable sign, symptom, or disease temporally associated with the treatment, whether or not caused by the treatment that results in death or is life-threatening or results in inpatient hospitalization or prolongation of existing hospitalization for more than 24 hours with a persistent or significant incapacity or substantial disruption of the ability to conduct normal life functions.

NRS: numeric rating scale.

In addition to the term “adverse events”, the term “side-effects” was used in 4 studies, “sequelae” was used in 4 studies, “complication” in 3 studies, “incident” in 2 studies and one study used the term “reaction”. New or worsened complaints or symptoms were described as adverse events in 18 studies. Twenty studies described adverse events as “unpleasant”, “unfavourable”, “unintended”, “unexpected” and/or “undesired responses”, and 5 studies as “untoward medical occurrences”. Common descriptors composing the adverse event definition statements were identified in the studies that provided a direct definition of an adverse event (n = 49). Specifically, causality was incorporated in the definition provided by 21 studies (e.g., “Adverse events are unexpected events […] without evidence of causality” [33]). Symptom severity was used to define adverse events in 20 studies (e.g., “Adverse events are moderate to severe […]” [34]). Symptom onset was included in the definition provided by 19 studies (e.g., “Adverse events were events that occurred within 24 hours following the treatment” [19]). Symptom duration was used as a descriptor in 10 studies (e.g., “[…] increased pain and/or stiffness of short duration” [35]). In general, studies including chiropractors defined adverse events as an “unpleasant reaction” or “new or worsened symptom” more often than studies including other professions.

Among the studies that provided a classification system (n = 50), 23 only described the end anchors of the classification (e.g., mild/minor and/or serious), 26 provided description of additional classification categories (e.g., moderate, severe, *etc*.) and 3 described a classification system not including severity (e.g., common and uncommon; preventable and not preventable). Common domains that were used to describe the severity classification categories included: intensity (e.g., “We classified adverse event intensity as NRS score of 1 to 3 = mild, score of 4 to 6 = moderate; and NRS score of 7 to 10 = severe” [47]); duration (e.g., mild = less than 24 hours, moderate = between 24 hours and 1 week, major = over 1 week [24]); functional impact (e.g., mild = function intact, moderate = function modified, major = function impaired [79]); and requirement of additional treatment (e.g., mild = no additional intervention, moderate = may need additional intervention, severe = required intensive intervention [55]). Overall, studies conducted in Asia classified adverse events according to the Spilker classification [90] more often than those conducted in other regions.

Among the 69 studies that provided a direct definition and/or classification system, 56 cited a reference or a source for the definition used, while 13 did not provide any reference or source. In total, 78 unique references were cited of which 55 were peer-reviewed publications and 23 were books, websites, online documents or other sources (Table 5). While most of the references were related to manual therapy (n = 46), studies and sources from other areas (e.g., oncology, pharmacology) and organizations (e.g., Agency for Healthcare Research and Quality, World Health Organization, National Patient Safety Agency) were also referenced (n = 29). Generally, studies including chiropractors cited Senstad et al. (1996a, 1996b, 1997) [36, 57, 91] more often than other professions; and studies including physiotherapists cited Carnes et al. (2010a) [23] and Carlesso et al. (2010, 2011) [16, 79] more often than other professions. Additionally, studies conducted in North America often referenced studies by Carnes et al. (2010a) [23] and Carlesso et al. (2010, 2011) [16, 79]; studies conducted in Europe often cited Carnes et al. (2010a, 2010b) [23, 92], Carlesso et al. (2010) [16], Senstad et al. (1997) [57], and Cagnie et al. (2004) [93]; and studies conducted in Asia referenced work from Spilker et al. (1991) [90] (S2 Table).

**Table 5:** References cited by studies providing a direct adverse event definition and/or classification system (n = 78).

Cited by # studies	Reference	
	Peer-reviewed articles (n = 55)	Books, websites and other sources (n = 23)
**17**	Carnes D, Mullinger B, Underwood M. Defining adverse events in manual therapies: a modified Delphi consensus study. Man Ther. 15:2–6.	
**10**	Carlesso LC, Macdermid JC, Santaguida LP. Standardization of adverse event terminology and reporting in orthopaedic physical therapy: application to the cervical spine. J Orthop Sports Phys Ther. 2010; 40:455–463	
**6**	Carlesso L, Cairney J, Dolovich L, Hoogenes J (2011) Defining adverse events in manual therapy: an exploratory qualitative analysis of the patient perspective. Manual Therapy 16: 440–446	
	Senstad O, Leboeuf-Yde C, Borchgrevink C. Frequency and characteristics of side effects of spinal manipulative therapy. Spine. 1997; 22:435–441	
**4**	Cagnie B, Vinck E, Beernaert A, Cambier D: How common are side effects of spinal manipulation and can these side effects be predicted? Man Ther 2004, 9(3):151–156	
	Carnes D, Mars TS, Mullinger B, Froud R, Underwood M. Adverse events and manual therapy: a systematic review. Man Ther 2010;15:355–63.	
	Edwards IR, Aronson JK. Adverse drug reactions: definitions, diagnosis, and management. Lancet. 2000;356:1255–9	
	Thiel HW, Bolton JE, Docherty S, Portlock JC: Safety of chiropractic manipulation of the cervical spine: a prospective national survey. Spine 2007, 32(21):2375–8	
		Spilker B. Interpretation of adverse reactions. In: Guide to clinical trials. New York: Raven Press, Ltd; 1992:565–587 / Spilker B. Quality of life and pharmacoeconomics in clinical trials. Philadelphia: Lippincott Williams & Wilkins. 1995;1312.
**3**	Barrett AJ, Breen AC. Adverse effects of spinal manipulation. J R Soc Med 2000;93:258–9	
	Ernst E: Adverse effects of spinal manipulation: a systematic review. J R Soc Med 2007, 100(7):330–338	
	Farrar JT, Young JP, LaMoreaux L, Werth JL, Poole RM. Clinical importance of changes in chronic pain intensity measured on an 11-point numerical pain rating scale. Pain 2001;94:149–58	
	Hurwitz E, Morgenstern H, Vassilaki M, Chiang L (2004) Adverse reactions to chiropractic treatment and their effects on satisfaction and outcomes among patients enrolled in the UCLA neck pain study. Journal of Manipulative and Physiological Therapeutics 27: 16–25.	
	Leboeuf-Yde C, Hennius B, Rudberg E, Leufvenmark P, Thunman M. Side effects of chiropractic treatment: a prospective study. J Manipulative Physiol Ther 1997;20:511–5.	
	Oliphant D: Safety of spinal manipulation in the treatment of lumbar disk herniations: a systematic review and risk assessment. J Manipulative Physiol Ther 2004, 27(3):197–210	
	Puentedura EJ, Landers MR, Cleland JA, Mintken PE, Huijbregts P, Fernandez-de-Las-Penas C. Thoracic spine thrust manipulation versus cervical spine thrust manipulation in patients with acute neck pain: a randomized clinical trial. J Orthop Sports Phys Ther. 2011;41:208–20	
	Rajendran D, Bright P, Bettles S, Carnes D, Mullinger B. What puts the adverse in ‘adverse events’? Patients’ perceptions of post-treatment experiences in osteopathy–a qualitative study using focus groups. Man Ther 2012;17(August (4)):305–11	
	Rubinstein SM, Leboeuf-Yde C, Knol DL, de Koekkoek TE, Pfeifle CE, van Tulder MW: The benefits outweigh the risks for patients undergoing chiropractic care for neck pain: a prospective, multicenter, cohort study. J Manipulative Physiol Ther 2007, 30(6):408–18.	
	Senstad O, Leboeuf-Yde C, Borchgrevink C. Side-effects of chiropractic spinal manipulation: types, frequency, discomfort and course. Scand J Primary Health Care. 1996; 14:50–53	
		Good clinical practice; ICH-GCP (E6), glossary art. 1.2. Available from: http://ichgcp.net/1-glossary [Internet]
		WHO Draft Guidelines for Adverse Event Reporting and Learning Systems. [http://www.who.int/patientsafety/events/05/Reporting_Guidelines. pdf]
**2**	Carlesso LC, Gross AR, Santaguida PL, Burnie S, Voth S, Sadi J. Adverse events associated with the use of cervical manipulation and mobilization for the treatment of neck pain in adults: a systematic review. Man Ther2010;15(October (5)):434–44	
	Eriksen K, Rochester RP, Hurwitz EL: Symptomatic reactions, clinical outcomes and patient satisfaction associated with upper cervical chiropractic care: a prospective, multicenter, cohort study. BMC Musculoskelet Disord 2011, 12:219	
	Pohlman K, O’Beirne M, Thiel H, et al. Development and validation of instruments to evaluate adverse events after spinal manipulation therapy. J Altern Complement Med. 2014;20(5). A49-A49	
	Puentedura EJ, March J, Anders J, Perez A, Landers MR, Wallmann HW, et al. Safety of cervical spine manipulation: are adverse events preventable and are manipulations being performed appropriately? A review of 134 case reports. J Man Manip Ther. 2012;20(2):66–74	
	Puentedura, E.J., O’Grady, W.H., 2015 Jul. Safety of thrust joint manipulation in the thoracic spine: a systematic review. J. Man. Manip. Ther. 23 (3), 154–161.	
	Rubinstein SM: Adverse events following chiropractic care for subjects with neck or low-back pain: do the benefits outweigh the risks? J Manipulative Physiol Ther 2008, 31(6):461–464	
	Rubinstein SM, Leboeuf-Yde C, Knol DL, de Koekkoek TE, Pfeifle CE, van Tulder MW: Predictors of Adverse Events Following Chiropractic Care for Patients with Neck Pain. J Manipulative Physiol Ther 2008, 31(2):94–103	
		Cancer therapy evaluation program, common terminology criteria for adverse events, version 4.0; 2009. Available from: http://ctep.cancer.gov/protocolDevelopment/electronicapplications/ctc.htm [Internet].
**1**	Basch E, Iasonos A, McDonough T, Barz A, Culkin A, Kris MG, et al. Patient versus clinician symptom reporting using the National Cancer Institute Common Terminology Criteria for Adverse Events: results of a questionnaire-based study. Lancet Oncol 2006;7(11):903e9.	
	Basch E, Jia X, Heller G, Barz A, Sit L, Fruscione M, et al. Adverse symptom event reporting by patients vs clinicians: relationships with clinical outcomes. J Natl Cancer Inst 2009; 101:1624e32	
	Bronfort et al. A randomized controlled clinical trial of rehabilitative exercise and chiropractic spinal manipulation for chronic neck pain. paper presented at: scientici symposium, World Chiropractic Congress; June 6–9, 1997; Tokyo, Japan.	
	Bronfort G, Evans R, Anderson AV, Svendsen KH, Bracha Y, Grimm RH: Spinal manipulation, medication, or home exercise with advice for acute and subacute neck pain: a randomized trial. Ann Intern Med 2012, 156(1 Pt 1):1–10	
	Bronfort G, Haas M, Evans R, Kawchuk G, Dagenais S: Evidence-informed management of chronic low back pain with spinal manipulation and mobilization. Spine J 2008, 8(1):213–225	
	Carlesso LC, Macdermid JC, Santaguida PL, et al. A survey of patient’s perceptions of what is “adverse” in manual physiotherapy and predicting who is likely to say so. J Clin Epidemiol. 2013;66(10):1184–1191.	
	Dvorak J, Orelli F. How dangerous is manipulation to the cervical spine? Case report and results of a survey. Manual Med 1985; 2:1–4.	
	Ernst, E., 2002 Apr 15, Manipulation of the cervical spine: a systematic review of case reports of serious adverse events, 1995–2001. Med. J. Aust. 176 (8), 376–380.	
	Gorrell LM, et al. The reporting of adverse events following spinal manipulation in randomized clinical trials-a systematic review. Spine J. 2016;16(9):1143–51.	
	Gouveia LO, Castanho P, Ferreira JJ. Safety of chiropractic interventions: a systematic review. Spine (Phila Pa 1976). 2009;34(11):E405-13.	
	Gross AR, Kay T, Hondras MA, Goldsmith CH, Haines T, Kennedy C, Peloso P 2002 Manual therapy for neck pain: A systematic review. Manual Therapy 7(3): 131–149.	
	Ioannidis J, Evans S, Gottzsche P, O’Neill R, Altman D, Schulz K. Moher D for the CONSORT group. Better reporting of harms in randomized trials: an extension of the CONSORT statement. Ann Intern Med 2004; 141:781e8.	
	Kranenburg HA, Schmitt MA, Puentedura EJ, et al. Adverse events associated with the use of cervical spine manipulation or mobilization and patient characteristics: A systematic review. Musculoskeletal Sci Pract. 2017;28:32–38	
	Leboeuf-Yde C, Axén I, Ahlefeldt G, Lidefelt P, Rosenbaum A, Thurnherr T: The types and frequencies of improved nonmusculoskeletal symptoms reported after chiropractic spinal manipulative therapy. J Manipulative Physiol Ther 1999, 22(9):559–64	
	Lord J, Littlejohns P. Evaluating healthcare policies: the case for clinical audit. BMJ 1997; 315:668–71.	
	Malone D, Baldwin N, Tomecek F, Boxell C, Gaede S, Covington C, et al. Complications of cervical spine manipulation therapy: 5 year retrospective study in a single group practice. Neurol Focus 2002;13.	
	Nielsen SM, Tarp S, Christensen R, Bliddal H, Klokker L, Henriksen M. The risk associated with spinal manipulation: an overview of reviews. Syst Rev. 2017;6(1):64	
	Paanalahti K, Holm LW, Nordin M, Asker M, Lyander J, Skillgate E. Adverse events after manual therapy among patients seeking care for neck and/or back pain: a randomized controlled trial. BMC Musculoskelet Disord. 2014; 15(1):77	
	Pohlman K, Carroll L, Tsuyuki R, Hartling L, Vohra S. Active versus passive adverse event reporting after pediatric chiropractic manual therapy: study protocol for a cluster randomized controlled trial. Trials. 2017;18(1):575.	
	Senstad O, Leboeuf-Yde C, Borchgrevink C. Predictors of side effects to spinal manipulative therapy. J Manipulative Physiol Ther. 1996; 19:441–445	
	Shafrir Y, Kaufman BA: Quadriplegia after chiropractic manipulation in an infant with congenital torticollis caused by a spinal cord astrocytoma. J Pediatr, 1992; 120:266–269	
	Skillgate E, Vingard E, Alfredsson L: Naprapathic manual therapy or evidence-based care for back and neck pain: a randomized, controlled trial. Clin J Pain 2007, 23(5):431–439.	
	Struewer J, Frangen TM, Ziring E, et al. Massive haemothorax after thoracic spinal manipulation for acute thoracolumbar pain. Orthop Rev (Pavia). 2013; 5:27	
	Terwee CB, Bot SDM, de Boer MR, van der Windt DA, Knol DL, DekkerJ, et al. Quality criteria were proposed for measurement properties of health status questionnaires. J Clin Epidemiol 2007; 60(1):34–42	
	Thoomes-de Graaf M, Thoomes E, Carlesso L, et al. Adverse effects as a consequence of being the subject of orthopaedic manual therapy training, a worldwide retrospective survey. Musculoskelet Sci Pract. 2017; 29:20–27.	
	Walker BF, Hebert JJ, Stomski NJ. Outcomes of usual chiropractic. The OUCH randomized controlled trial of adverse events. Spine. 2013; 38:1723–9	
	Walshe K. Adverse events in health care: issues in measurement. Qual Health Care. 2000; 9(1):47–52.	
	White C. Doctors mistrust systems for reporting medical mistakes. BMJ 2004; 329:12–3.	
	Williams K. Patients will be able to report drugs’ side effects. BMJ 2004; 328:1095.	
	Woloshynowych M, Neale G, Vincent C. Case record review of adverse events: a new approach. Qual Saf Health Care 2003; 12:411–5.	
		AHRQ’s patient safety initiative: building foundations, reducing risk. Appendix 1. Patient safety terms and definitions; 2003. Avail-able from: http://www.ahrq.gov/research/findings/final-reports/pscongrpt/psiniapp1.html [Internet]
		Davies JM, Hebert PC, Hoffman C. The Canadian patient safety dictionary. Edmonton: Canadian Patient Safety Institute; 2003. The National Patient Safety Agency (NPSA). Seven steps to patient safety for primary care. London, UK: NPSA; 2005
		Department of Health. An organisation with a memory. London: Stationery Office; 2000.
		Department of Health. Building a safer NHS for patients. London: Stationery Office; 2001.
		Greenman PE: Principles of Manual Medicine, ed 2. Baltimore: Williams and Wilkins, 1996, pp 99–103
		Griffin FA, Resar RK. IHI global trigger tool for measuring adverse events. IHI Innovation Series white paper, 2nd ed. Cambridge, MA: Institute for Healthcare Improvement; 2009
		Kleynhans AM. Complications of and contraindications to spinal manipulative therapy. In: Haldeman S, ed. Modern Developments in the Practice and Principles of Chiropractic. Norwalk, CT: Appleton-Century-Crofts, 1980:359–82.
		Livingston MC: Spinal manipulation causing injury. A three year study. Clin Orthop 81:82–86, 1971.
		Maigne R. Orthopedic medicine: A new approach to vertebral manipulations. 3rd ed. Liberson WT, ed., trans. Springfield, IL: Charles C Thomas, 1972:153–5
		MedWatch. The FDA safety information and adverse event reporting program. What is a serious adverse event? Available from: http://www.fda.gov/safety/medwatch/howtoreport/ucm053087.htm [Internet]
		NPSA. Exploring incidents—improving safety. A guide to root cause analysis from the NPSA. In: www.npsa.uk/rcatoolkit/course/iindex.htm.
		SafteyNet. Available from: http://care.ualberta.ca/SafetyNET.aspx [Internet]
		Secker-Walker J, Taylor-Adams S. Clinical incident reporting. In: Vincent C, editor. Clinical risk management. Enhancing patient safety. London: BMJ Publishing Group; 2001. p. 419–38
		The Texas Chiropractic Association: Texas Guidelines for Chiropractic Quality Assurance and Practice Parameters. Gaithersburg, MD: Aspen Publishers, 1994, pp 167–177
		Van Liew D. Using information systems to support risk management. In: Wilson J, Tingle J, editors. Clinical risk modification. A route to clinical governance. Oxford: Butterworth Heinemann; 1999. p. 66–85.
		World Health Organisation 2009. In: Clinical Safety Data Management: Definitions and Standards for Expedited Reporting (Ed. by E. M. Agency), pp. 58e59. Available at: http://www.ema.europa.eu/docs/en_GB/document_library/Regulatory_and_procedural_guideline/2009/10/WC500004443.pdf. Accessed on 10 October, 2013.
		WHO Patient Safety. Conceptual framework for the international classification for patient safety, version 1.1: technical annex 2. Geneva, Switzerland: World Health Organization (WHO); 2010
		Zhao P. Spinal health handbook. Beijing: Popular Science Press; 2010. p. 63–6.
		Zhao P. Personalized attention on lumbar disc herniation. Beijing: Popular Science Press; 2009. p. 40–5.

#### Studies with an indirect adverse event definition and/or classification system

The studies that provided an indirect definition of an adverse event and/or classification system (n = 29) were published between 1996 and 2021, mostly from North America (n = 14) and The United Kingdom (n = 6). Eleven studies focused on spinal and/or peripheral joint manipulation, 1 focused on mobilization and 17 included both techniques. Most were conducted in an academic institute (n = 14) and provided manipulation or mobilization to the spine (n = 19).

Study designs included: reviews (n = 8), trials (n = 6), surveys (n = 5), observational studies (n = 4), clinical practice guidelines (n = 2), case reports/series (n = 2), protocol (n = 1) and qualitative study (n = 1). Of these 29 studies, 4 provided an indirect definition of an adverse event only, 10 provided a classification system only, and 15 provided both an indirect definition and classification system (Tables 6–8).

**Table 6:** Studies providing indirect adverse event definition only (n = 4).

Authors, year, citation	Study design	Definition
**Giles et al. 2003** [98]	Observational Study	Events requiring remedial treatment; **minor** transient increase in spinal pain symptoms that did not qualify as an adverse event
**Hay et al. 2005** [94]	Trial	“An exacerbation of pain after the initial assessment’’
**de Oliveira et al. 2013** [32]	Trial	“Did your symptoms get worse after this treatment?”
		“Are you feeling any different symptom after this treatment?”
**Satpute et al. 2020** [99]	Case Reports/Series	“There were no adverse events during or after each treatment session in terms of dizziness, increase in pain intensity, or induction of other symptoms.’’

**Table 7:** Studies providing indirect adverse event classification system only (n = 10).

Authors, year, citation	Study design	Classification system
**Barrett et al. 2000** [100]	Survey	“**Hardly any** discomfort, **mild** discomfort, **moderate** discomfort, **severe** discomfort, **worst possible** discomfort—one hour, one day and two days after SMT’’
**Meeker et al. 2002** [101]	Review	“. . . onset within 4 hours of the procedure, disappearing within 24 hours’’; **’’Serious** complication cases including fatalities, major impairments’’
		Mentions **severe, serious**
**Anderson-Peacock et al. 2005** [102]	Clinical Practice Guideline	**Non-treatment** adverse events–not associated with a treatment modality, but that occur in the clinical setting;
		**Unforseen-treatment** adverse events–associated with a treatment modality, but not a known or observable risk factor;
		**Forseen-treatment** adverse events–associated with a treatment modality and predicted by an observable risk factor
Cleland et al. 2007 [97]	Trial	“Subjects. . . were asked to report the time of onset (categorized as ≤24 hours or >24 hours), the duration (categorized as ≤24 hours or >24 hours), and the severity (scored on a scale of 1–4, where 1 = **light** to 4 = **severe**) of the symptoms’’; Mentions **mild, moderate, serious**
**Dagenais et al. 2010** [103]	Review	**Minor**, temporary, self-limiting (side-effects)
		**serious**; last between several hours and a few days
**Carlesso et al. 2010** [104]	Review	“The adverse events were initially grouped into **major**—death, stroke or permanent neurological deficits and **minor**—transient neurological symptoms, increased neck pain/stiffness, headache, radiating pain, fatigue or other’’
**Yin et al. 2014** [105]	Review	**Mild, minor, moderate, medium, serious**; appeared within 4–24 hours of treatment; disappeared within 24 hours; rated ≤ NRS regarding severity
**Coulter et al. 2019** [106]	Review	Minor; typically transient
**Funabashi et al. 2020** [107]	Survey	Variation in terms of frequency and severity, ranging from the more frequent **minor/benign** adverse events to **rare and serious** adverse events
**Zhang et al. 2021** [108]	Protocol	“Shedding criteria: 1) **intolerable** adverse reactions; 2) **serious** adverse reactions; 3) the patients’ pain continued to increase, which proved that trial participation was not suitable; 4) the patient’s health may be damaged (for example, **serious** complications)’’

NRS: numeric rating scale; SMT = spinal manipulative therapy.

**Table 8:** Studies providing indirect adverse event definition & classification system (n = 15).

Authors, year, citation	Study design	Definition	Classification system
**Senstad et al. 1996b** [91]	Survey	**Benign** and short-lasting discomfort	“The patient was asked for the degree of pain (four choices ranging from “**minor**” to “**unbearable**”). The chiropractor then asked three questions: 1) “How soon after the treatment did the reaction start?”, 2) “How long did the reaction last?”, and 3) “Could you carry out your usual work?” ’’
**Ernst et al. 2001** [109]	Review	“Transient exacerbation of symptoms’’;	“**Light**, **moderate**, **fairly severe** and **more than that**’’; **’’Serious** injury’’; **’’Mild, moderate, definitely unpleasant, and unbearable**’’; ’’The onset of symptoms was mostly on the day of the intervention. or the day after’’; **’’Moderate, slight’’**; ’’Very noticeable. . . . associated with a reduction in the ability to work’’; ’’. . . .had disappeared within 24 hours’’
		’’Unpleasant reactions’’;	
		’’Adverse response, no matter how minor or fleeting’’;	
		’’Any discomfort (other than presenting symptoms) experienced after treatment’’	
**Cagnie et al. 2004** [93]	Survey	Unpleasant reactions	“The patient was asked to report the type of reaction, time of onset, duration and severity of symptoms, whether any reaction had caused difficulty in performing daily activities and how they felt 48hr after treatment’’
**Murphy et al. 2006** [110]	Case Reports/Series	“Transient increase in pain to **serious** complications’’	**Major** complications; transient adverse reactions; short-lived, transient
**Bronfort et al. 2008** [96]	Review	**Benign** temporary side effects; typically does not interfere with activities of daily living	Typically does not interfere with activities of daily living; occurred within 4 hours; mild-to-moderate severity; disappeared the same day
**Brantingham et al. 2009** [111]	Trial	“**Serious** adverse reactions (e.g., defined as persistent severe knee stiffness, swelling and/or pain)’’	**Mild, serious**
**Langworthy et al. 2010** [112]	Survey	"**Serious** adverse reaction to cervical spine manipulation resulting in stroke or other significant neurological damage"	**Serious**
**Bronfort et al. 2012** [113]	Trial	Expected, typical of treatment; transient in nature, requiring little or no change to activity levels	**Serious / nonserious**
**Rajendran et al. 2012** [114]	Qualitative Study	Loss of function, particularly if it had an impact on work or daily activities; unexpected, in nature or intensity; unacceptable	**Major**, **moderate**, or **minor** and “**not adverse**’’, were classified according to duration, severity and seriousness/acceptability
**Page et al. 2014** [115]	Review	Pain persisting longer than 2 hours after treatment or more disability the next morning	**Mild, minor, serious**
**Bronfort et al. 2014** [95]	Trial	Different type of pain;	**Mild, moderate, self-limiting, serious**
		Increased symptom severity;	
		Increased difficulty with activities	
**Bussieres et al. 2018** [116]	Clinical Practice Guideline	Undesirable effects/outcome; transient increase in pain	Undesirable; **serious**
**Morris et al. 2018** [117]	Observational Study	Injury or loss of normal function; increased pain or new pain complaints. Therapeutic measures were/were not required	“All complications were classified as an ‘unexpected pain exacerbation’ ’’
**Peters et al. 2019** [118]	Observational Study	**Non-serious** adverse events	“Intensity and duration linked to the following: (1) aggravation of complaints in treated area; (2) radiating pain to an upper extremity; (3) headache; 4) stiffness in the treated area; (5) tiredness; (6) dizziness or light-headedness; (7) nausea; 8) ringing in the ears; (9) confusion or disorientation; (10) cramps; (11) blurred vision; (12) weakness in the limbs; (13) vomiting; and (14) any other symptom not defined by any of the previous categories’’
**Mabry et al. 2020** [119]	Observational Study	Events that have reached a patient and are subsequently categorized by the level of harm endured by the patient	“**Mild, transient, and self-limiting** adverse events’’

Indirect definitions commonly referred to adverse events as “new or worsening symptoms” (e.g., “One adverse reaction (an exacerbation of pain after the initial assessment) was recorded.” [94]). The most commonly used categories used to classify adverse events were words such as “minor”, “mild”, “moderate”, “serious” and “severe” (e.g., “[…] adverse events were mild to moderate, self-limiting, and reported by 30% of patients […]” [95]). Common domains that were used to indirectly describe the severity classification categories included: onset (e.g., “Most of these AEs *[adverse events]* occurred within 4 hours of SMT *[spinal manipulative therapy]*” [96]); duration (e.g., “If the subjects indicated that they had experienced any side effect, then they were asked to report […], the duration (categorized as ≤24 hours or ≥24 hours) […]” [97]); and/or action taken (e.g., “There was no record of interrupted treatment due to side effects” [91]).

Among the 29 studies that provided an indirect definition and/or classification system, 14 cited a reference or a source for the definition used, while 15 did not. In total, 27 unique references were cited among which 24 were peer-reviewed publications and 3 were books, websites, online documents or other sources (Table 9). Most of the references were related to manual therapy, including spinal and peripheral joint manipulation and mobilization, (n = 20); however, other areas (e.g., aviation) and sources (e.g., clinical practice guideline, dictionary) were also cited (n = 7).

**Table 9:** References cited by studies providing an indirect adverse event definition and/or classification system (n = 27).

Cited by # studies	References	
	Peer-reviewed articles (n = 24)	Books, websites and other sources (n = 3)
**6**	Senstad O, Leboeuf-Yde C, Borchgrevink C. Frequency and Characteristics of side effects of spinal manipulative therapy. Spine 1997;22(4):435–41.	
**4**	Leboeuf-Yde et al. Side effects of chiropractic treatment: a prospective study. JMPT 1997; 20(8):511–515	
**3**	Cagnie B, Vinck E, Beernaert A, Cambier D. How common are side effects of spinal manipulation and can these side effects be predicted? Manual Therapy 2004;9(3):151–156.	
**2**	Carnes D, Mullinger B, Underwood M. Defining adverse events in manual therapies: a modified Delphi consensus study. Manual Therapy 2010;15(1):2e6.	
	Senstad O, Leboeuf-Yde C, Borchgrevink CF. Side-effects of chiropractic spinal manipulation: types, frequency, discomfort and course. Scand J Prim Health Care 1996;14(1):50–53	
		Dictionary Oxford: Oxford University Press; 1999 & 2006
**1**	Carlesso LC, Cairney J, Dolovich L, Hoogenes J. Defining adverse events in manual therapy: an exploratory qualitative analysis of the patient perspective. Man Ther 2011;16(5):440e6.	
	de Campos TF. Low back pain and sciatica in over 16s: assessment and management NICE Guideline [NG59]. J Physiother. 2017;63(2):120.	
	Ernst E. Prospective investigations into the safety of spinal manipulation. Journal of Pain and Symptom Management, vol. 21, no. 3, pp. 238–242, 2001.	
	Fish D, Kretzmann H, Brantingham JW, Globe G, Korporaal C, Moen J. A randomized clinical trial to determine the effect of combining a topical capsaicin cream and knee joint mobilization in the treatment of osteoarthritis of the knee. J Am Chiropr Assoc 2008;45:8–23.	
	Hebert JJ, Stomski NJ, French SD, Rubinstein SM. Serious adverse events and spinal manipulative therapy of the low back region: a systematic review of cases. J Manip Physiol Ther. 2015;38(9):677–691.	
	Hurwitz et al. Manipulation and mobilization of the cervical spine. A systematic review of the literature. Spine 1996; 21(15):1746–59	
	Hurwitz, H. Morgenstern, M. Vassilaki, and L. Chiang, “Adverse reactions to chiropractic treatment and their effects on satisfaction and clinical outcomes among patients enrolled in the UCLA Neck Pain Study,” Journal of Manipulative and Physiological Therapeutics, vol. 27, no. 1, pp. 16–25, 2004.	
	Hurwitz EL, Morgenstern H, Vassilaki M, et al. Frequency and clinical predictors of adverse reactions to chiropractic care in the UCLA Neck Pain Study. Spine (Phila Pa 1976). 2005; 30(13):1477–1484	
	Long A, Esmonde L, Connolly S. A typology of negative responses: a case study of shiatsu. Complement Ther Med 2009;17:168e75.	
	Nadareishvili Z, Norris JW. Stroke from traumatic arterial dissection. The Lancet 1999;354:158–159.	
	Nixdorf D: Current standards of material risk. JCCA 1990, 34(2):87–89	
	Paanalahti K, Holm LW, Nordin M, et al. Adverse events after manual therapy among patients seeking care for neck and/or back pain: a randomized controlled trial. BMC Musculoskelet Disord. 2014;15:77.	
	Paige NM, Miake-Lye IM, Booth MS, et al. Association of spinal manipulative therapy with clinical benefit and harm for acute low back pain: systematic review and meta-analysis. JAMA. 2017;317(14):1451–1460.	
	Rajendran D, Mullinger B, Fossum C, Collins P, Froud R. Monitoring self-reported adverse events: a prospective, pilot study in a UK osteopathic teaching clinic. Int J Osteopath Med 2009;12(2):49e55.	
	Rivett DA, Milburn P. A prospective study of complications of cervical spine manipulation. J Man Manip Ther 1996;4:166–170.	
	Rubinstein SM, Leboeuf-Yde C, Knol DL, de Koekkoek TE, Pfeifle CE, van Tulder MW. Predictors of adverse events following chiropractic care for patients with neck pain. J Manipulative Physiol Ther 2008;31:94e103.	
	Swait G, Finch R. What are the risks of manual treatment of the spine? A scoping review for clinicians. Chiropr Man Ther. 2017;25:1–15.	
	Walker BF, Hebert JJ, Stomski NJ, et al. Outcomes of usual chiropractic; harm (OUCH) randomised controlled trial of adverse events. Spine (Phila Pa 1976). 2013;38:1723–9.	
		Dvorak J, Kranzlin P, Muhleman D, Walchli B. Musculoskeletal complications. In: Haldeman S, editor. Principles and practice of chiropractic. Norwalk: Appleton & Lange, 1992:549–77
		SECAF SotAF: Air Force Instruction 44–119: Medical Quality Operations. In: Department of the Air Force; 2011:290.

## Discussion

This study mapped the scientific literature discussing the definition of adverse events and their classification systems following spinal and peripheral joint manipulation and mobilization for musculoskeletal conditions in an adult population. Based on the 98 included studies, heterogeneous adverse event definitions and classification systems were identified. This empirical evidence of heterogeneity highlights the need for international and interprofessional consensus on a standardized definition and classification system so that patient safety practices for spinal and peripheral joint manipulation and mobilization can be more homogeneous, facilitating synthesis of findings and outcomes and, consequently, improving patient care.

### Definition components

Despite adverse events following spinal and peripheral joint manipulation and mobilization being investigated since the 1990s, a clear definition and classification system has yet to be established. Our findings highlight that even the terms used to refer to adverse events vary, ranging from “side effects” to “symptomatic responses” to “harms”, “complications”, “adverse response/reaction/effects/events/ experience”, among others [36, 42, 45, 48, 66, 71, 91]. Given the importance of this topic and the increased focus of healthcare on patient safety [13], it is surprising that the standardization of terms, definition and classification system have not yet been established. This, in turn, could have contributed to the lack of adverse event reporting systems within professions that use joint manipulation and mobilization interventions. An established reporting system that accurately collects the number of spinal and peripheral joint manipulations and mobilizations delivered [120] would allow more precise estimations of the incidence of adverse events following these interventions and potential contributing factors to such events could be investigated.

Our findings identified descriptors commonly used to define adverse events and classification systems: causality, symptom severity, onset and duration, and action taken. Establishing causality between the delivery of a manual intervention and an adverse event is challenging. This relationship/association has long been a discussion within manual therapy [23, 121, 122], other healthcare arenas [123–127] and the overall field of patient safety [18, 128]. Recently, a tool to assess causality of adverse events associated with any therapeutic interventions has been developed that could assist with this complex issue [129]. In addition to being one of many components of adverse event definitions identified in this study, this standardized tool to assess causality may assist with the identification of predisposing factors which, in turn, could contribute to the development of prevention and mitigation strategies of similar adverse events [130]. Therefore, a standardized operational definition for what constitutes an adverse event is needed to allow for the identification of adverse events across professions and this tool could be used to assess the causality of accurately identified adverse events.

Symptom severity (or intensity), onset and duration, and action taken (e.g., medication use, seeking unplanned medical care) were descriptors commonly used to define both what constitutes an adverse event and describe the classification system categories (Tables 2–4). For example, symptom severity was observed within adverse event definitions when it included the worsening and aggravation of a symptom (e.g., increased pain). Additionally, adverse events were classified as minor (or mild), moderate or major (or severe) based on the symptom duration, with minor (or mild) adverse events being short-duration (e.g., less than 24 hours) and major (or severe) adverse events being long term. Although different studies used different thresholds (e.g., considering short duration 24 or 48 hours), these descriptors were observed in most included studies.

Overall, in healthcare, adverse events have been classified based on the intervention (e.g., vaccine adverse event), anatomical location (e.g., eye adverse event), severity (e.g., serious adverse event), or causality (e.g., causal adverse event) [18]. However, specific classifications within medical areas have been developed to better characterize adverse events, contributing to advancements within patient safety by enabling better synthesis of information [131–134]. For example, Kaafarani et al. (2014) proposed that the classification of intraoperative adverse events to range from Class I (injury requiring no repair with the same procedure) to Class VI (intraoperative) [134]. Kaufman (2016) identified that not all adverse drug reactions fit into the previously established types A (predictable) and B (novel responses) and proposed the addition of type C (continuing), type D (delayed use) and type E (end of use) reactions [133]. Therefore, developing a standardized classification specific for adverse events following spinal and peripheral joint manipulation and mobilization could provide a common language for all professions that use these interventions and facilitate identification, reporting and communication about adverse events, promoting interprofessional learning and contributing to advancing patient safety.

### Multidisciplinarity

Although adverse events following spinal and peripheral joint manipulation and mobilization have been the focus of several studies, these often include one profession (e.g., chiropractic, naprapathy, osteopathy, physiotherapy, *etc*.) [19, 31, 47, 60]. Given the number of professions using these manual therapy interventions, it is possible that the inter-professional knowledge exchange related to definitions and classification of adverse events was limited as each profession focused on their individual (siloed) professional communities rather then the intervention at large. Indeed, this review identified that included studies tended to cite references that were published by authors in the same profession. Although communication across health-related professions has been observed to be well-established and a common practice among academic communities [135, 136], joint manipulation and mobilization providers have been described to present an unique culture related to patient safety [137]. Specifically, divergent intra- and inter-profession beliefs, overlapping scopes of practice and perceived business competition may prevent interprofessional communications focused on adverse events following these interventions [137]. However, in order to advance joint manipulation and mobilization safety initiatives, enhanced interprofessional communications and collaborations are not only possible but fundamental. We have attempted to address this issue by establishing an international, multidisciplinary working group investigating adverse event definition and classification systems across all professions using joint manipulation and mobilization. Another example includes the international framework for risk assessment of cervical artery dysfunction [138], which included a multidisciplinary research team.

Furthermore, in 2010a, Carnes and colleagues conducted a multidisciplinary Delphi study with the aim to seek an expert consensus definition for adverse events applicable to all professions that use manual therapy [23]. Similarly, Carlesso et al. (2011) explored how patients receiving manual therapy from different professions defined adverse events [79]. These are two of the few multidisciplinary studies, including different professions (i.e., chiropractic, osteopathy, physiotherapy) that use spinal and peripheral joint manipulation and mobilization and were referenced by 19% and 7% of the 98 included studies in this scoping review, respectively. This percentage of referencing is slightly lower than the average 20%-35% interprofessional referencing in medical sciences [136] and could potentially explain, at least partially, the heterogenous adverse event definition and classification systems observed in this study. Although Carnes et al. (2010a) did not achieve consensus on a succinct adverse event definition, a proposed classification system was clearly determined and described [23]. Remarkably, even though this work was published over a decade ago, the definition of an adverse event and their classification systems remained noticeably heterogeneous in the manual therapy literature, including in studies published after Carnes et al. (2010a). This reinforces the possibility of limited interprofessional knowledge exchange related to this specific topic, and the importance of the broad dissemination of results going beyond individual professions, as well as efforts from all professions to enhance interprofessional, topic-related knowledge, rather than profession-specific.

### Geography

The heterogeneity in adverse event definitions and classification systems identified in this study could potentially be due to the fact that spinal and peripheral joint manipulation and mobilization are interventions commonly used by different professionals located in different geographical locations [139–141]. Variation in professions’ scope of practice and regulations between continents, countries and even regional jurisdictions could lead providers to use their own definition for an adverse event, based on their local practices and regulations [139, 142]. Additionally, the emergence of litigation most commonly related to serious adverse events following manipulation leading to significant disability, such as vertebral artery dissection, cauda equina syndrome, *etc*., may have contributed to the development of local definitions [11, 21, 80, 104]. As the number of serious and life-threatening adverse events reports following manipulation increased, so did the number of malpractice lawsuits against professionals who use these interventions [143–145]. Consequently, legal courts, lawyers and malpractice insurers were likely compelled to develop local definitions in order to process and rule on such cases. Given that any practising provider is vulnerable to experiencing malpractice lawsuits against them, they may feel bound to these local definitions to be consistent with the environment in which they practice.

Additionally, given the diverse geographical locations in which spinal and peripheral joint manipulation and mobilization are used, cultural differences and their influence on individual beliefs and behaviours could also be a potential contributor to the adverse event definition and classification system heterogeneity found in this study [146]. Culture refers to values, norms, and codes that collectively shape the beliefs, attitudes, and behavior of a group [147]. Indeed, the impact of culture on health has been widely investigated as better understanding cultural contexts advances the knowledge of inter-personal roles, connections, and relationships (whether positive or negative), as well as allowing the understanding of how individuals are shaped and their health [147–150]. Consequently, cultural differences can play an important role in how adverse events after these interventions are defined and classified and may have a significant contribution to the heterogeneity identified in this study.

This review identified trends in citations where specific continents used specific references more often in comparison to other continents. Besides demonstrating a potential limitation in knowledge exchange across geographical locations, this finding highlights the paucity of studies related to this topic from some parts of the world, including Africa and South America. Therefore, including these continents when developing a standardized adverse event definition and classification system is of great importance not only to take into consideration geographical and cultural particularities, but also to support the development of investigations related to this topic in these locations.

### Future studies

Based on these findings, an e-Delphi study will be conducted to establish a standardized adverse event definition and classification system that can be prospectively used across multiple professions [30]. This has the potential to greatly advance patient safety as it would provide a standardized framework for data to be collected and synthesized in an uniform manner. This would then provide all stakeholders of spinal and peripheral joint manipulation and mobilization interventions a comprehensive patient safety profile for the adult population with musculoskeletal conditions. Insights gained from this profile could assist with the formation and streamlining of clinical guidelines and further research capacities.

### Strengths and limitations

Strengths of this study include the involvement of an interprofessional research group with clinical and methodological expertise, and development of the protocol *a priori* for transparency. Additionally, this review was not limited by country or profession; therefore, our findings are representative and transferable to an international and interprofessional audience.

Although the search included several potential terms related to adverse events, it is possible that potentially relevant studies that used alternate terms to describe adverse events were not captured. The search was also limited to studies published in English, Italian and Portuguese languages; potentially relevant studies published in other languages (such as German, French, Dutch, etc.) were not captured. Additionally, adverse event definitions provided by included studies were categorized into “direct” and “indirect”. Although this categorization was clearly defined (i.e., direct definition provided a clear statement of what was considered an adverse event; indirect definition indicated what was considered an adverse event without a clear statement [e.g., provided the question asked to participants during the study]), it is not an established categorization and contains some level of subjectivity.

## Conclusion

Findings identified that a vast array of terms, definitions and classification systems for adverse events following spinal and peripheral joint manipulation and mobilization have been published. Within this array of literature, there was no one standardized adverse event definition or classification system for adverse events following these interventions that is commonplace and widely used. This suggests that establishing a consensus on standardized terms, definitions and classification systems for adverse events related to these interventions is urgently needed and could advance strategies to enhance patient safety for all professions who deliver these interventions.

## Supporting information

S1 Checklist Preferred Reporting Items for Systematic reviews and Meta-Analyses extension for Scoping Reviews (PRISMA-ScR) checklist. (DOCX)

S1 Table The initial search strategy developed for Ovid MEDLINE. (PDF)

S2 Table Citation by geographical location. (DOCX)
