# Supplementary material for: Definition and classification for adverse events following spinal and peripheral joint manipulation and mobilization: A scoping review
Source: PLoS One. 2022 Jul 15;17(7):e0270671. doi: 10.1371/journal.pone.0270671 (PMC9286262; doi:10.1371/journal.pone.0270671)
Supplement: S1 Table — (PDF) [file pone.0270671.s002.pdf]

Friday, February 12, 2021 8:50:53 AM

#	Query	Limiters/Expanders	Last Run Via	Results
S8	S5 AND S6	Limiters - Language: English, Italian, Portuguese<br>Expanders - Apply equivalent subjects<br>Search modes - Find all my search terms	Interface - EBSCOhost Research Databases<br>Search Screen - Advanced Search<br>Database - MEDLINE	1,516
S7	S5 AND S6	Expanders - Apply equivalent subjects<br>Search modes - Find all my search terms	Interface - EBSCOhost Research Databases<br>Search Screen - Advanced Search<br>Database - MEDLINE	1,620
S6	(MH "Classification+") OR TI (classif* OR defin* OR index* OR framework OR standards OR consisten* OR scal* OR grad* OR rating OR cod* OR polic* OR consent* OR consensus OR konsensus OR agree* OR assess* OR standard* OR terminology OR taxonom* OR terms OR severity) OR AB (classif* OR defin* OR index* OR framework OR standards OR consisten* OR scal* OR grad* OR rating OR cod* OR polic* OR consent* OR consensus OR konsensus OR agree* OR assess* OR standard* OR terminology OR taxonom* OR terms OR severity)	Expanders - Apply equivalent subjects<br>Search modes - Find all my search terms	Interface - EBSCOhost Research Databases<br>Search Screen - Advanced Search<br>Database - MEDLINE	9,682,008
S5	S3 OR S4	Expanders - Apply equivalent subjects<br>Search modes - Find all my search terms	Interface - EBSCOhost Research Databases<br>Search Screen - Advanced Search<br>Database - MEDLINE	3,131
S4	(MH "Musculoskeletal Manipulations/AE") OR (MH "Manipulation, Osteopathic/AE") OR (MH "Manipulation,	Expanders - Apply equivalent subjects<br>Search modes - Find all my search terms	Interface - EBSCOhost Research Databases<br>Search Screen - Advanced Search<br>Database - MEDLINE	956

	Chiropractic/AE") OR (MH "Chiropractic/AE") OR (MH "Manipulation, Orthopedic/AE") OR (MH "Manipulation, Spinal/AE")			
S3	S1 AND S2	Expanders - Apply equivalent subjects<br>Search modes - Find all my search terms	Interface - EBSCOhost Research Databases<br>Search Screen - Advanced Search<br>Database - MEDLINE	2,549
S2	(MH "Patient Safety+") OR TI (adverse or complication* or contraindic* or harm or harmful* or harming or harmed or hazard* or "side effect*" or ((effect* or event* or outcome* or reaction* or consequen*) N3 (undesirabl* or injurious* or negativ*))) OR AB (adverse or complication* or contraindic* or harm or harmful* or harming or harmed or hazard* or "side effect*" or ((effect* or event* or outcome* or reaction* or consequen*) N3 (undesirabl* or injurious* or negativ*))) or TI (safe*) or AB (safe* N9 (patient* or treatment* or therap* or manipulat* or procedure* or outcome* or intervention* or method* or practic*))	Expanders - Apply equivalent subjects<br>Search modes - Find all my search terms	Interface - EBSCOhost Research Databases<br>Search Screen - Advanced Search<br>Database - MEDLINE	2,388,896
S1	(MH "Musculoskeletal Manipulations") OR (MH "Manipulation, Osteopathic") OR (MH "Manipulation, Chiropractic") OR (MH "Chiropractic") OR (MH "Manipulation, Orthopedic") OR (MH "Manipulation, Spinal") OR TI (chiropra* OR quiropra*) OR TI ((manip* OR mobiliz* OR mobilis* OR adjustment*) N5 (musculoskeletal* or orthopaedic or orthopedic or osteopath*)) OR TI ((manip*	Expanders - Apply equivalent subjects<br>Search modes - Find all my search terms	Interface - EBSCOhost Research Databases<br>Search Screen - Advanced Search<br>Database - MEDLINE	20,552

OR manual) N1 (therap* or treat* OR "physical therap*")) OR AB (chiropra* OR quiropra*) OR AB ((manip* OR mobiliz* OR mobilis* OR adjustment*) N5 (musculoskeletal* or orthopaedic or orthopedic or osteopath*)) OR AB ((manip* OR manual) N1 (therap* or treat* OR "physical therap*"))
