# Supplementary material for: Definition and classification for adverse events following spinal and peripheral joint manipulation and mobilization: A scoping review
Source: PLoS One. 2022 Jul 15;17(7):e0270671. doi: 10.1371/journal.pone.0270671 (PMC9286262; doi:10.1371/journal.pone.0270671)
Supplement: S2 Table — (DOCX) [file pone.0270671.s003.docx]

**S2 Table.** Citation by geographical location

| **Reference** | **Continent** |
| --- | --- |
| Carnes D, Mullinger B, Underwood M. Defining adverse events in manual therapies: a modified Delphi consensus study. Man Ther. 15:2-6. | Asia: 0  Australia: 1  Europe: 8  North America: 8 |
| Carlesso LC, Macdermid JC, Santaguida LP. Standardization of adverse event terminology and reporting in orthopaedic physical therapy: application to the cervical spine. J Orthop Sports Phys Ther. 2010;40:455-463 | Asia: 0  Australia: 1  Europe: 3  North America: 6 |
| Carlesso L, Cairney J, Dolovich L, Hoogenes J (2011) Defining adverse events in manual therapy: an exploratory qualitative analysis of the patient perspective. Manual Therapy 16: 440– 446 | Asia: 0  Australia: 1  Europe: 2  North America: 3 |
| Senstad O, Leboeuf-Yde C, Borchgrevink C. Frequency and characteristics of side effects of spinal manipulative therapy. Spine. 1997; 22:435-441 | Asia: 0  Australia: 1  Europe: 3  North America: 2 |
| Cagnie B, Vinck E, Beernaert A, Cambier D: How common are side effects of spinal manipulation and can these side effects be predicted? Man Ther 2004, 9(3):151–156 | Asia: 0  Australia: 1  Europe: 3  North America: 0 |
| Carnes D, Mars TS, Mullinger B, Froud R, Underwood M. Adverse events and manual therapy: a systematic review. Man Ther 2010;15:355-63. | Asia: 0  Australia: 0  Europe: 3  North America: 1 |
| Edwards IR, Aronson JK. Adverse drug reactions: definitions, diagnosis, and management. Lancet. 2000;356:1255–9 | Asia: 0  Australia: 2  Europe: 2  North America: 0 |
| Thiel HW, Bolton JE, Docherty S, Portlock JC: Safety of chiropractic manipulation of the cervical spine: a prospective national survey. Spine 2007, 32(21):2375-8 | Asia: 0  Australia: 1  Europe: 2  North America: 1 |
| Spilker B. Interpretation of adverse reactions. In: Guide to clinical trials. New York: Raven Press, Ltd; 1992:565-587 / Spilker B. Quality of life and pharmacoeconomics in clinical trials. Philadelphia: Lippincott Williams & Wilkins. 1995;1312. | Asia: 4  Australia: 0  Europe: 0  North America: 0 |
| Barrett AJ, Breen AC. Adverse effects of spinal manipulation. J R Soc Med 2000;93:258-9 | Asia: 0  Australia: 0  Europe: 2  North America: 1 |
| Ernst E: Adverse effects of spinal manipulation: a systematic review. J R Soc Med 2007, 100(7):330–338 | Asia: 1  Australia: 0  Europe: 2  North America: 0 |
| Farrar JT, Young JP, LaMoreaux L, Werth JL, Poole RM. Clinical importance of changes in chronic pain intensity measured on an 11-point numerical pain rating scale. Pain 2001;94:149-58 | Asia: 0  Australia: 0  Europe: 2  North America: 1 |
| Hurwitz E, Morgenstern H, Vassilaki M, Chiang L (2004) Adverse reactions to chiropractic treatment and their effects on satisfaction and outcomes among patients enrolled in the UCLA neck pain study. Journal of Manipulative and Physiological Therapeutics 27: 16–25. | Asia: 0  Australia: 2  Europe: 1  North America: 0 |
| Leboeuf-Yde C, Hennius B, Rudberg E, Leufvenmark P, Thunman M. Side effects of chiropractic treatment: a prospective study. J Manipulative Physiol Ther 1997;20:511-5. | Asia: 0  Australia: 1  Europe: 1  North America: 1 |
| Oliphant D: Safety of spinal manipulation in the treatment of lumbar disk herniations: a systematic review and risk assessment. J Manipulative Physiol Ther 2004, 27(3):197-210 | Asia: 1  Australia: 0  Europe: 1  North America: 1 |
| Puentedura EJ, Landers MR, Cleland JA, Mintken PE, Huijbregts P, Fernandez-de-Las-Penas C. Thoracic spine thrust manipulation versus cervical spine thrust manipulation in patients with acute neck pain: a randomized clinical trial. J Orthop Sports Phys Ther. 2011;41:208–20 | Asia: 0  Australia: 0  Europe: 1  North America: 2 |
| Rajendran D, Bright P, Bettles S, Carnes D, Mullinger B. What puts the adverse in ‘adverse events’? Patients’ perceptions of post-treatment expe-riences in osteopathy – a qualitative study using focus groups. Man Ther2012;17(August (4)):305–11 | Asia: 0  Australia: 0  Europe: 1  North America: 2 |
| Rubinstein SM, Leboeuf-Yde C, Knol DL, de Koekkoek TE, Pfeifle CE, van Tulder MW: The benefits outweigh the risks for patients undergoing chiropractic care for neck pain: a prospective, multicenter, cohort study. J Manipulative Physiol Ther 2007, 30(6):408-18. | Asia: 0  Australia: 1  Europe: 1  North America: 1 |
| Senstad O, Leboeuf-Yde C, Borchgrevink C. Side-effects of chiropractic spinal manipulation: types, frequency, discomfort and course. Scand J Primary Health Care. 1996; 14:50-53 | Asia: 0  Australia: 0  Europe: 2  North America: 1 |
| Good clinical practice; ICH-GCP (E6), glossary art. 1.2. Available from: http://ichgcp.net/1-glossary [Internet] | Asia: 0  Australia: 0  Europe: 2  North America: 1 |
| WHO Draft Guidelines for Adverse Event Reporting and Learning Systems. [http://www.who.int/patientsafety/events/05/Reporting_Guidelines. pdf] | Asia: 0  Australia: 0  Europe: 2  North America: 1 |
